# Advances in Understanding the Physiological and Molecular Responses of Sugar Beet to Salt Stress

**DOI:** 10.3389/fpls.2019.01431

**Published:** 2019-11-06

**Authors:** Xiaoyan Lv, Sixue Chen, Yuguang Wang

**Affiliations:** ^1^School of Life Science and Technology, Harbin Institute of Technology, Harbin, China; ^2^Key Laboratory of Sugar Beet Genetic Breeding of Heilongjiang Province, Heilongjiang University, Harbin, China; ^3^Department of Biology, Genetics Institute, Plant Molecular and Cellular Biology Program, University of Florida, Gainesville, FL, United States

**Keywords:** sugar beet, salt stress, salt tolerance, proteomics, transcriptomics, metabolomics

## Abstract

Soil salinity is a major environmental stress on crop growth and productivity. A better understanding of the molecular and physiological mechanisms underlying salt tolerance will facilitate efforts to improve crop performance under salinity. Sugar beet is considered to be a salt-tolerant crop, and it is therefore a good model for studying salt acclimation in crops. Recently, many determinants of salt tolerance and regulatory mechanisms have been studied by using physiological and ‘omics approaches. This review provides an overview of recent research advances regarding sugar beet response and tolerance to salt stress. We summarize the physiological and molecular mechanisms involved, including maintenance of ion homeostasis, accumulation of osmotic-adjustment substances, and antioxidant regulation. We focus on progress in deciphering the mechanisms using ‘omic technologies and describe the key candidate genes involved in sugar beet salt tolerance. Understanding the response and tolerance of sugar beet to salt stress will enable translational application to other crops and thus will have significant impacts on agricultural sustainability and global food security.

## Introduction

Soil salinity is a major environmental stress that affects agricultural production worldwide. Globally, about 960 million hectares of arable land are affected by excess salt levels (Munns and Tester, 2008; Deinlein et al., 2014; Jha et al., 2019). The adverse effects of an annual salinity increase of ∼1-2% are exacerbated by global climate change, poor irrigation practice, and improper fertilizer utilization (Zhu, 2016). Furthermore, the human population is expected to reach 9.6 billion by 2050 (Shabala et al., 2015). The additional food required to feed this growing population has imposed great pressures on existing natural resources. Therefore, improving plant salt tolerance is useful for improving agricultural production and food security. Salinity causes ionic imbalance, osmotic stress, and secondary stresses, *e.g*., oxidative stress. These stresses have a substantial impact on crop life from seed germination to vegetative growth to reproduction and seed filling. Plant responses to salt stress include a decrease in leaf expansion, stomatal closure, inhibition of photosynthesis, and reduced biomass (Zhang and Shi, 2013). On the other hand, plants have evolved a large number of physiological and biochemical processes to adapt to salt stress. In general, the adaptive responses of plants to salt stress can be grouped into three categories: osmotic stress tolerance, ion exclusion, and tissue tolerance to salinity (Yang and Guo, 2018a).

Sugar beet (*Beta vulgaris* L.) belongs to the order *Caryophyllales*, which lies at the basal taxa of core dicots. It is an important root crop in the world for sugar production, where its tap roots are used. The world production of sugar from sugar beet in 2018 was approximately 42 million metric tons, accounting for nearly 30% of the world sugar supply (FAO). In addition to its use in the sugar industry, it is a major source for animal feed and the production of bioethanol as renewable energy (Magaña et al., 2011). A wild ancestor of sugar beet is *Beta maritima* L. (sea beet), which grows naturally along the Atlantic coasts of Western Europe and the coasts of Mediterranean countries (Doney et al., 1999). To survive in these habitats, sea beet has developed structural and physiological strategies to regulate the distribution of salt and other solutes and to increase water content (Daoud et al., 2003). Many sugar beet cultivars inherited the salt tolerance trait from their ancestor and are considered salt-tolerant glycophytes. Sugar beet can tolerate up to 500 mM sodium chloride (NaCl) for seven days without losing viability (Yang et al., 2012). Moreover, it has been found that when the electrical conductivity (EC) of soil reached 7.0 dS m^-1^ (**≈**67 mM NaCl), the yield of sugar beet was not affected (Marschner, 1995). With the completion of the genome sequencing of sugar beet (Dohm et al., 2013), it is considered to be an excellent crop model for studying salt tolerance mechanisms.

Previous studies have mainly focused on the physiological responses of sugar beet to salt stress. Recently, more and more studies have focused on elucidating the molecular mechanisms of salt tolerance using different ‘omics tools, such as RNA sequencing (Lv et al., 2018; Skorupa et al., 2019), proteomic analysis (Wakeel et al., 2011a; Yang et al., 2012; Yu et al., 2016), and metabolomic analysis (Hossain et al., 2017a). These large-scale studies have rapidly delivered new knowledge and important insights into the molecular processes of salt tolerance in sugar beet (**Table 1**). However, to date, there has been no critical review of the recent advances toward understanding the molecular and physiological mechanisms of sugar beet salt tolerance. This review article aims to fill this knowledge gap by providing an overview of recent progress made in the field of sugar beet salt tolerance, including its physiological and molecular mechanisms as revealed by ‘omics technologies and the genes targeted for genetic improvement and molecular breeding for crop salt tolerance.

**Table 1:** Major ‘omic studies of salt stress tolerance in sugar beet using different technological platforms.

Tissue/Species	Salt treatment	Technique	Key findings	Reference
Leaves and roots/*Bv*M14	0/400 mM NaCl	SSH	Identified differentially expressed genes in *Bv*M14 under salt stress	Ma et al., 2017
Leaves and roots/*Bv*M14	0/200/400 mM NaCl	RNA-SEQ	Identified differentially expressed genes in *Bv*M14 under salt stress	Lv et al., 2018
Leaves/*B. maritime*	0/150/300 mM NaCl	RNA-SEQ	Investigated transcriptome response to acute salt stress imposed to excised leaves of sea beet	Skorupa et al., 2016
Leaves/*B. maritime* and *B. vulgaris*	0/300 mM NaCl	RNA-SEQ	Revealed alterations in beet leaf transcriptome during acclimation to stress and response to shock, and identified salinity-related and genotype-specific traits in the patterns of gene expression in leaves of sea beet and sugar beet.	Skorupa et al., 2019
Leaves and roots/*Bv*M14	0/200/400 mM NaCl	2D-DIGE/iTRAQ	Analyzed salt-responsive proteins in *Bv*M14 plants under salt stress	Yang et al., 2012; Yang et al., 2013
Leaves and roots/*B. vulgaris* (T510, salt-tolerant) and *B. vulgaris* (S210, salt- sensitive)	0/280 mM NaCl	iTRAQ	Identified differentially changed proteins between the sensitive and tolerant cultivars of sugar beet, and provided a list of potential markers for the further engineering of salt tolerance in crops	Wang et al., 2019
Leaves/*Bv*M14	0/200/400 mM NaCl	iTRAQ	Analyzed the *Bv*M14 membrane proteome under salt stress	Li et al., 2015
Leaves/*Bv*M14	0/200/400 mM NaCl	Label-free quantitative proteomics	Profiled changes in the *Bv*M14 phosphoproteome induced by salt stress	Yu et al., 2016
Leaves/*B. vulgaris*	0/300 mM NaCl	GC-MS	Investigated metabolic adaptations of sugar beet to salt stress through GC-MS of whole leaf tissues and chloroplasts	Hossain et al., 2017a

SSH, suppression subtractive hybridization; RNA-SEQ, RNA Sequencing; iTRAQ, isobaric tag for relative and absolute quantification; 2D-DIGE, two-dimensional difference gel electrophoresis.

## Effects of Salt Stress

Although sugar beet is salt-tolerant compared to other crops, it is sensitive to salinity at the germination and early seedling stages (Kaffka and Hembree, 2004;Sadughi et al., 2015). Water uptake and availability is essential for seed germination and early seedling growth. Salt stress causes a significant reduction in water availability, decreases the mobilization of stored reserves, and affects the structural organization of proteins, leading to poor germination (Sadughi et al., 2015). However, the usual long lag time of sugar beet seeds in salinized soil may initiate seed priming (AboKassem, 2007;Sadeghian and Yavari, 2010), enabling germination at a salinity level of 12 dS m^-1^ (**≈**115 mM NaCl), which does not significantly affect seed germination compared to control conditions (Jafarzadeh and Aliasgharzad, 2007; Khayamim et al., 2014). When the salinity level reached 16 dS m^-1^ (**≈**150 mM NaCl) or a combination of different salts compositions (Mg_2_SO_4_/NaCl/Na_2_SO_4_/CaCl_2_) were imposed, sugar beet seed germination was found to be significantly inhibited (Jafarzadeh and Aliasgharzad, 2007). Presumably, decreased osmotic potential caused by high salt concentration inhibits water imbibition in seeds. The establishment of sugar beet seedlings is more susceptible to salinity inhibition than is seed germination. For example, the root length is significantly decreased at an EC of 4 dS m^-1^ (**≈**38 mM NaCl) (Jafarzadeh and Aliasgharzad, 2007). It was also reported that the hypocotyl length and number of normal seedlings were negatively correlated with the increase of salt level. Therefore, long roots and hypocotyls and a low percentage of abnormal seedlings (as defined by the International Seed Testing Association, 1985) may be used as indexes for identifying salt-tolerant genotypes of sugar beet (Khayamim et al., 2014).

Chlorophyll is the major pigment in plant photosynthesis and is responsible for absorbing and transforming light energy. Chlorophyll content is thus an important physiological indicator of plant salt-stress damage. The content of chlorophyll in sugar beet was decreased by 38.4% at 280 mM NaCl (EC**≈**33 dS m^-1^) but did not change under mild salinity (EC = 5.5 dS m^-1^ **≈**55 mM NaCl.) (Hajiboland et al., 2009; Wang et al., 2017). In addition, the net photosynthesis rate and stomatal conductance showed similar change trends to those of the chlorophyll level under the above two salt-stress conditions. Rubisco, which is directly involved in CO_2_ fixation, is a determining factor for carbohydrate accumulation in plants. Its activity was obviously decreased under salt stress in comparison with control (Hossain et al., 2017b; Wang et al., 2017). These adverse effects eventually lead to decreases in the leaf area, growth, and root yield of sugar beet.

Salt stress also affects the uptake and accumulation of mineral nutrients in sugar beet. For example, high salinity increases the phosphate content of sugar beet in a dose-dependent manner. Excessive accumulation of phosphorus may lead to phosphorus poisoning, growth retardation, and necrosis in plants (Zhou et al., 2008). Nitrate reductase (NR) is a key enzyme in plant nitrogen acquisition and is responsible for the synthesis of nitric oxide (NO), a key signaling molecule in plant cells (Chamizo-Ampudia et al., 2017). NR activity was found to be significantly inhibited by NaCl in young and old leaves of sugar beet. The degree of decrease in the enzyme activity was greater with increasing salt concentration (Ghoulam et al., 2002). The reduction of NR activity was mainly due to the excess accumulation of salt in the cytoplasm. However, direct links between salt and NR regulation are not known. Another report showed that salinity induced lower nitrogen assimilation in sugar beet at the end of its growth season compared to earlier in the season (Hajiboland et al., 2009). Although this situation caused sugar beet to have a lower dry matter and shoot mass, the weight of the storage root and the sugar content increased significantly because of a change in the partitioning of organic materials in favor of the roots.

## Primary Mechanisms of Salt Tolerance in Sugar Beet

### Accumulation of Osmotic Adjustment Substances

Osmotic stress is the primary form of stress suffered by plants when subjected to salt stress. Osmotic adjustment is vital for the alleviation of the osmotic imbalances caused by salt stress and for maintaining cell turgor (Liang et al., 2018). It involves cellular solute accumulation in response to a decrease in the water potential of the environment. Although sugar beet is sensitive to salinity at seed germination and early seedling stage (see the previous section), established plants exhibit a high osmotic adjustment capacity, as reflected by the accumulation of organic and inorganic osmolytes under salt stress (Katerji et al., 1997; Wu et al., 2015a). Several studies have found that glycinebetaine (GB), choline, free amino acids, and proline accumulated in sugar beet leaves with increasing NaCl concentrations in the growth medium (Ghoulam et al., 2002; Yamada et al., 2009; Wu et al., 2013a). High levels of GB (>20 mmol g FW^-1^) in young leaves of sugar beet were detected under normal growth conditions (Yamada et al., 2009). In contrast, the contents of GB in old leaves, cotyledons, hypocotyls, and roots were low. GB is primarily synthesized in old leaves and is translocated into young leaves (Yamada et al., 2009). As a result, high accumulation of GB was observed in the new leaves of sugar beet, especially under salt-stress conditions (Russell et al., 1998; Yamada et al., 2009). GB clearly plays a key role in the osmotic adjustment of sugar beet (Waditee et al., 2007). GB is synthesized *via* a two-step reaction catalyzed by choline monooxygenase (CMO) and betaine aldehyde dehydrogenase (BADH) (Chen and Murata, 2002). CMO catalyzes the first step, which is the rate-limiting step in GB synthesis (Hibino et al., 2002). Antisense *BvCMO* transgenic sugar beets with suppressed levels of *Bv*CMO protein exhibited decreased GB synthesis and became more susceptible to salt stress compared to wild-type plants (Yamada et al., 2015). On the other hand, transplastomic tobacco plants over-expressing the *BvCMO* gene accumulated GB and exhibited increased tolerance to salt stress (Zhang et al., 2008). Thus, *BvCMO* has the potential to be used in genetic engineering to improve plant salt stress tolerance. GB was found to be transported by proline transporters using a transmembrane proton gradient (Yamada et al., 2011). Whether the transport and distribution of GB in different plant tissues affects plant salt tolerance needs further investigation.

Proline is another important osmolyte in plant cells, and its cellular levels can be used as a physiological index of plant salt stress tolerance in many species (Per et al., 2017; De la Torre-González et al., 2018). However, the importance of proline accumulation in sugar beet under salt stress for osmotic adjustment is still under debate. It is noteworthy that shoot proline concentrations were significantly higher in salt-tolerant cultivars than in salt-sensitive cultivars under control or salt-stress conditions (Ghoulam et al., 2002;Wu et al., 2013a). It was speculated that high proline contents in the salt-tolerant sugar beet genotypes could be induced by cellular demand for osmotic adjustment and membrane stabilization. However, from a quantitative point of view, the contribution to osmotic adjustment by the accumulated proline appeared to be small compared to GB. Furthermore, inorganic salt ions may play a more important role than proline in osmotic adjustment. It was reported that sugar beet seedlings accumulate high levels of ions such as Na^+^, K^+^, and Cl^-^ in their shoots, which are involved in effective osmotic adjustment under salt-stress conditions (Wu et al., 2015b).

### Redox Regulation of Salt Tolerance

Salt stress induces the accumulation of cellular reactive oxygen species (ROS), such as superoxide radical (O^2−^), hydrogen peroxide (H_2_O_2_), and hydroxyl radical (·OH), that are generated by plant photosynthetic and respiratory electron transport systems, xanthine oxidases, and NADPH oxidases. Usually, cellular ROS levels are regulated by both non-enzymatic antioxidants and enzymatic antioxidants (Apel and Hirt, 2004; Baxter et al., 2014;Ben Rejeb et al., 2015). Interestingly, H_2_O_2_ levels in sugar beet were found to be lower under long-term salinity than under control conditions (Hossain et al., 2017b). This result is quite different from the responses of other crop species, which exhibit salt-stress-induced ROS accumulation (Mittler et al., 2004). It is speculated that sugar beet is efficient in adjusting the cellular redox environment under salinity. The expression of *superoxide dismutases (SOD)* (e.g., *Cu-Zn-SOD*, *Mn-SOD*, and *Fe-SOD*) and *peroxiredoxins (Prx)* were increased under salt stress (Hossain et al., 2017b). The induced expression of these antioxidant-related genes helps to remove accumulations of ROS. In the meantime, transcription of *respiratory burst oxidase homolog* (*RBOH*) isoforms, the major ROS-generating *NADPH oxidases*, were significantly suppressed under salinity. These mechanisms of maintaining low ROS accumulation help to mitigate oxidative stress and facilitate normal cellular metabolism for the growth and development of sugar beet under salt stress.

Changes in antioxidant enzymes involved in salt stress tolerance have also been investigated in the cultivated sugar beet *B. vulgaris* and its wild salt-tolerant relative *B. maritime*. The activities of SOD, peroxidase (POX), ascorbate peroxidase (APX), catalase (CAT), and glutathione reductase (GR) in *B. maritime* were obviously higher than in *B. vulgaris* (Bor et al., 2003). APX is one of the major members in the anti-oxidation system that scavenges the excess H_2_O_2_ caused by salt stresses (Guan et al., 2015). APX can convert H_2_O_2_ into H_2_O with ascorbic acid (ASA) as the electron donor. Interestingly, the expression of *BvAPX* was up-regulated under salt stress in the leaves of both *B. maritime* and *B. vulgaris*. However, a much longer duration of salt stress was required to induce *APX* gene expression in the salt-tolerant *B. maritime* compared to in the salt-sensitive varieties (Dunajska-Ordak et al., 2014). The highly efficient osmotic regulation in *B. maritime* may account for the delayed induction of *APX* expression. Peroxidase (POX) is another well-known antioxidant enzyme that protects plant cells from oxidative damage, and its enzymatic activity was induced in the roots of sugar beet under stress conditions (Pradedova et al., 2014). Recently, it was reported that the level of *POX* gene transcription under salt stress was related to the elevated levels of acetylation in H3K9 and H3K27 sites in sugar beet (Yolcu et al., 2016). In *B. vulgaris* and *B. maritime*, the acetylation patterns were significantly different. Unlike the cultivated *B. vulgaris*, the main acetylation site of the salt-tolerant wild species is H3K9. These studies indicate that the antioxidant system plays a key role in determining sugar beet salt tolerance, and epigenetics appears to regulate the antioxidant system at the transcriptional level.

### Maintaining Ion Homeostasis

When plants are exposed to a saline environment, Na^+^ can enter cells through non-selective cation channels and K^+^ transporters (Ward et al., 2003). Thus, maintaining ion homeostasis is imperative for plants to adapt to salt stress (Nadeem et al., 2019). Usually, plants eliminate excessive Na^+^ from the cytosol *via* the plasma membrane or tonoplast Na^+^/H^+^ antiporters (NHX) to maintain an optimal cytosolic Na^+^ level (Zhu, 2003; Olías et al., 2009). These Na^+^/H^+^ antiporters use the electrochemical gradient of protons across the tonoplast or plasma membrane to move Na^+^ into the vacuole or outside the cell, respectively (Blumwald, 2000). It has been suggested that the activity of vacuolar Na^+^/H^+^ antiporters is significantly different in salt-tolerant versus salt-sensitive plants. Compared with salt-sensitive plants, *NHX* transcription in salt-tolerant plants is much more strongly induced (Yokoi et al., 2002; Gong et al., 2010). Furthermore, tonoplasts of salt-tolerant sugar beet exhibited high tonoplast NHX activity, which was directly related to the salt stress tolerance of *B. vulgaris* cell cultures (Blumwald and Poole, 1987; Wu et al., 2019). The transcription of *BvNHX1* was significantly increased under salt stress. This pattern of increase was consistent with elevated BvNHX1 protein and vacuolar NHX activity (Xia et al., 2002). Another study showed that the sugar beet *BvNHX1* gene was modulated by MYB transcription factor(s), which were responsible for activating its expression upon salt exposure (Adler et al., 2010). Interestingly, overexpression of tonoplast *NHXs* in sugar beet was shown to improve salt stress tolerance (Yang et al., 2005; Liu et al., 2010). The transgenic sugar beet accumulated high levels of potassium and low levels of salt in the roots. Furthermore, the transgenic sugar beet exhibited higher soluble sugar content and yield in storage roots under saline conditions than the wild type (Liu et al., 2015). Simultaneously with the activation of vacuolar *BvNHX* under salt stress, the transcription of vacuolar H^+^ pump *V-H* *^+^* *-ATPase* was also enhanced (Kirsch et al., 1996). This coordinated regulation of both *NHX* and *V-H* *^+^* *-ATPase* in sugar beet constitutes an efficient mechanism underlying vacuolar salt sequestration and salt tolerance in sugar beet (Kirsch et al., 1996).

The restriction of K^+^-efflux is another important mechanism underlying the salt tolerance of sugar beet. Plasma membrane (PM) H^+^-ATPase was found to be involved in restricting K^+^ efflux under salt-stress conditions (Sun et al., 2010;Shabala et al., 2016). A previous study compared the effect of salt stress on PM H^+^-ATPase activity in salt-sensitive maize and salt-tolerant sugar beet (Wakeel et al., 2011b). Although high concentrations of salt can inhibit PM H^+^-ATPase activity, the activity in the salt-tolerant sugar beet was less affected than that in maize. At low concentrations of salt, the PM H^+^-ATPase activity in sugar beet was not affected, while an obvious decrease of activity was detected in maize. These results indicate that PM H^+^-ATPase in sugar beet is relatively stable under salt stress and can maintain a high level of cellular K^+^. In addition, apoplastic pH, which is determined by the H^+^-ATPases, was not affected in sugar beet under salt stress (Kirsch et al., 1996; Wakeel et al., 2010). According to the acid-growth theory, lower apoplastic pH allows extension growth by increasing cell wall extensibility. Therefore, the relatively stable PM H^+^-ATPase under salt stress ensures an acid-growth condition for sugar beet.

## ‘Omics Technologies for Discovering the Genes, Proteins and Metabolites Involved in Salt Tolerance

To date, many studies have focused on utilizing ‘omics tools to explore salt-tolerance mechanisms in plants. The ‘omics tools include genomics, transcriptomics, proteomics, and metabolomics, which allow large-scale discovery of candidate genes, proteins, and metabolites involved in plant salt stress tolerance (Sun et al., 2017; Lei et al., 2018). Recently, several important genes, proteins and metabolites related to salt tolerance in sugar beet have been reported (**Table 1**). Here we describe how the ‘omics results help to improve understanding of the salt-tolerance mechanisms in sugar beet.

### Transcriptomic Study of Salt Tolerance in Sugar Beet

**Figure 1:**
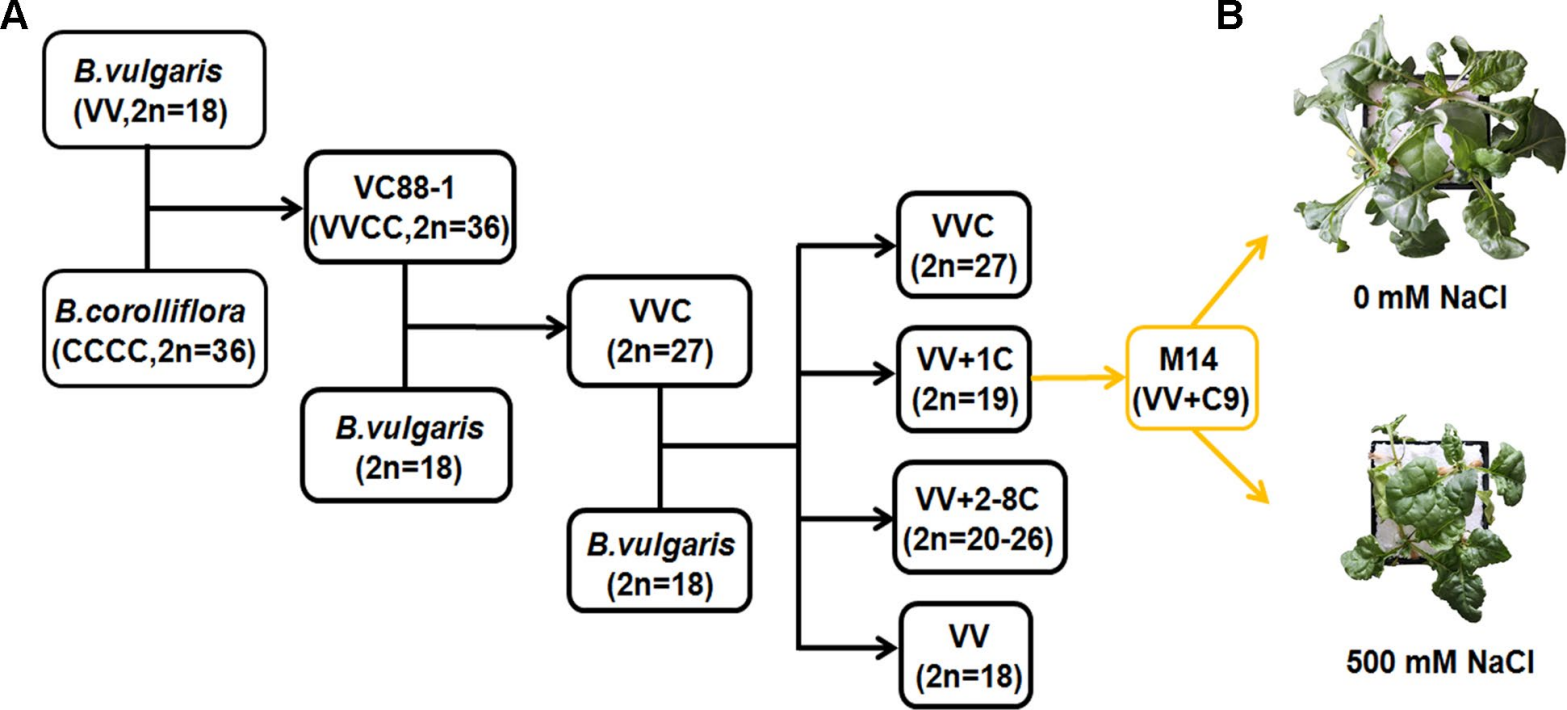
Generation of sugar beet M14 and its phenotype under salt stress. **(A)** Breeding process of sugar beet M14 (*B. vulgaris* genome plus chromosome No. 9 from *B. corolliflora*). **(B)** Growth status of sugar beet M14 under salt stress for seven days.

In the early days, the polymerase chain reaction (PCR)-based suppression subtractive hybridization (SSH) method was adopted to compare gene expression patterns between ‘tester’ and ‘driver’ populations (Wang et al., 2005). A number of differentially expressed genes related to salt-stress tolerance were identified in different plant species by the SSH technique (Baldwin and Dombrowski, 2006; Ouyang et al., 2006). In our previous work, a single chromosome from *B. corolliflora* was introduced into the cultivated *B. vulgaris* through the traditional crossing of distant species. One of the hybrid lines, *Bv*M14, containing chromosome 9 of *B. corolliflora* was obtained (**Figure 1**). It showed characteristics of apomixis and tolerance to salt stress (Li et al., 2010). *Bv*M14 tolerated up to 500 mM NaCl treatment (**Figure 1**). The SSH method was applied to explore changes in the transcriptional profiles of the *Bv*M14 plants under salinity (Ma et al., 2017). Tester and driver cDNAs were synthesized from *Bv*M14 root and leaf mRNA extracted from control and salt-stress plants. A total of 36 differentially expressed genes were identified and annotated in *Bv*M14 roots and leaves under salt stress. Most of the genes were involved in metabolism and photosynthesis. For example, one of the differentially expressed genes, *S-adenosylmethionine synthetase 2* (*BvM14-SAMS2*), is an important enzyme in the synthesis of S-adenosylmethionine (SAM), a precursor of polyamines. It was suggested that polyamines may be involved in determining plant responses to abiotic or biotic stresses. As expected, transgenic *Arabidopsis* plants overexpressing *BvM14-SAMS2* exhibited strong salt and H_2_O_2_ tolerance compared to the wild type (Ma et al., 2017). Another salt-responsive gene, *BvM14-cystatin*, identified by the SSH method, was also isolated from sugar beet and overexpressed in *Arabidopsis*. The transgenic plants showed enhanced salt tolerance (Wang et al., 2012). Taken together, these studies showed that the SSH technique was useful for identifying key genes involved in sugar beet salt tolerance in the early days.

Recently, high-throughput sequencing tools developed to monitor gene expression patterns have made it possible to systemically explore how plants cope with salt stress (Sun et al., 2013; Bushman et al., 2016). In order to determine a broad spectrum of genes involved in *Bv*M14 salt tolerance, comparative transcriptomics analysis was performed to identify differentially expressed genes in the leaf and root samples of *Bv*M14 seedlings at 0, 200 and 400 mM NaCl conditions (Lv et al., 2018). GO and KEGG enrichment analyses found that a large proportion of the differentially expressed genes were concentrated in redox balance, signal transduction, and protein phosphorylation. In addition, the differentially expressed genes of *Bv*M14 under salt stress were very different in the leaves and in the roots. This result indicates that the *Bv*M14 roots and leaves have different adaptation mechanisms for coping with salt stress. Moreover, the genes involved in the ROS scavenging system, such as *APX*, *SOD*, *thioredoxin (TRX)*, *glutathione S-transferase (GST)*, *monodehydroascorbate reductase (MDAR)*, and *glycolate oxidase (GOX)*, showed significant differences in sugar beet under salt stress compared to control. This result indicates that the plant antioxidant system plays an important role in regulating sugar beet salt tolerance.

As sea beet (*B. maritima*) displays elevated salt-tolerance compared to the cultivated beet (*B. vulgaris*), a study also investigated the transcriptomic responses to acute salt stress imposed to excised leaves of sea beet. Differentially expressed genes involved in osmoprotection, molecular chaperoning, and redox protein synthesis were identified and may play a key role in determining salt tolerance in sea beets (Skorupa et al., 2016). Recently, another study using RNAseq was published that explored transcriptional patterns related to salt responses in sugar beet (*B. vulgaris*) and its wild ancestor sea beet (Skorupa et al., 2019). Two kinds of salt treatment strategies were applied: either a gradual increase in salt concentration (salt-stress) or sudden exposure to salinity (salt-shock). Interestingly, sugar beet exhibited more significant transcriptomic changes to maintain homeostasis than sea beet. In addition, salt shock induced greater transcriptomic changes than salt stress, and salt shock led to a larger number of up-regulated genes compared to salt stress. Moreover, this study also confirmed that sugar beet inherited salt-tolerance traits from sea beet and that bHLH transcription factors are candidate regulators of salt-stress responses in sugar beet (Skorupa et al., 2019).

### Proteomic Study of Salt Tolerance in Sugar Beet

Although some of the salt-responsive genes have been identified in sugar beet using high-throughput RNA sequencing, transcriptomic data does not often correlate with the results of proteomic data due to posttranscriptional, translational, and posttranslational regulations (Chen and Harmon, 2006; Li et al., 2015; Walley et al., 2016). Therefore, it is imperative to employ proteomics to investigate global protein level changes under salt stress. Previously, two sugar beet cultivars with contrasting salt tolerance were selected to compare their proteomes’ response to salt using an isobaric tag for a relative and absolute quantification (iTRAQ)-based proteomic approach (Wang et al., 2019). This study indicated that salt-sensitive and -tolerant sugar beet cultivars exhibited different changes in proteomic profiles under salt stress. Several proteins involved in protein modification, the tricarboxylic acid cycle, cell wall synthesis, and reactive oxygen species scavenging showed differential changes between the sensitive and tolerant cultivars, indicating that these pathways may participate in the salt tolerance of sugar beet. Some potential markers for further engineering of salt tolerance in sugar beet have been identified, such as late embryogenesis abundant (LEA) proteins, abscisic acid-stress ripening protein 1 (ASR1), and S-adenosylhomocysteine synthase. Furthermore, salt-responsive characteristics of the salt-tolerant sugar beet *Bv*M14 were studied under 0, 200, and 400 mM NaCl conditions using two-dimensional difference gel electrophoresis (2D-DIGE) and gel-free iTRAQ approaches (Yang et al., 2012; Yang et al., 2013). Differentially expressed proteins involved in photosynthesis, respiration, the antioxidant system, methionine metabolism, and GB synthesis were all increased in the roots and leaves of *Bv*M14. The results indicated that enhancement of photosynthesis and methionine metabolism, accumulation of compatible organic solutes and antioxidative enzymes, and increase in ion uptake/exclusion were the main regulatory mechanisms underlying salt tolerance in *Bv*M14. One of the differentially expressed proteins, *Bv*M14-glyoxalase I, was found to exhibit a two-fold increase in leaves in response to salt treatment. *BvM14-glyoxalase I* was ubiquitously expressed in different tissues of *Bv*M14 and displayed increased levels under salt, mannitol, and oxidative stresses (Wu et al., 2013b). To investigate the functions of *BvM14-glyoxalase I*, it was constitutively expressed in *Nicotiana tabacum*. The transgenic plants showed marked tolerance to methylglyoxal, salt, mannitol, and H_2_O_2_. Another differential protein in *Bv*M14, S-adenosylmethionine decarboxylase (*Bv*M14-SAMDC), is a key rate-limiting enzyme involved in plant polyamine synthesis. Recently, the gene encoding the *Bv*M14-SAMDC protein was cloned from *Bv*M14 leaves (Ji et al., 2019). When *BvM14-SAMDC* was overexpressed, increased levels of spermidine (Spd) and spermin (Spm) and high activities of antioxidant enzymes were observed compared to in the wild type. Interestingly, expression levels of *RbohD* and *RbohF*, which play a role in ROS production, were significantly decreased in the transgenic plants (Ji et al., 2019). This study indicates that biosynthesis of Spm and Spd contributes to sugar beet salt stress tolerance through enhanced antioxidant activities and decreased ROS generation.

Plant cells have extensive membrane systems that play a key role in regulating responses and adaptation to salt stress. To understand the essential functions of membrane systems, an iTRAQ-based comparative proteomic study was conducted using microsomes of sugar beet *Bv*M14 plants under control and salt-stress conditions (Li et al., 2015). This study revealed that plasma membrane ATPase 11 and vacuolar ATPase subunit H were increased in response to salt stress. These proteins were involved in generating a proton gradient for ion transport across the plasma membrane and vacuolar membrane, respectively. Increasing the levels and activity of the ATPases may be an effective strategy for Na^+^ sequestration and osmotic adjustment under salt stress.Protein phosphorylation is one of the most widespread post-translational regulations in plant cell signaling under salt stress (Hubbard and Cohen, 1993; Tanou et al., 2012). Changes in the phosphoproteome of *Bv*M14 plants under short-term salt stress were analyzed using label-free quantitative proteomics (Yu et al., 2016). Several key kinases were found to exhibit significant changes under salt stress, including mitogen-activated protein kinases (MAPKs), 14-3-3s, receptor kinases, and calcium-dependent protein kinases (CDPKs). In addition, the phosphorylation of peroxiredoxin and several other proteins was a novel and intriguing discovery. Furthermore, sugar beet casein kinase 2 (CK2) was induced by salt stress and has been proved critical to salt tolerance in *Saccharomyces cerevisiae* (Kanhonou et al., 2001). With the exception of phosphorylation, other post-translational modifications have rarely been studied in sugar beet and should be an exciting subject for future research.

### Metabolomic Study of Salt Tolerance in Sugar Beet

Global metabolic changes can reflect protein activities and physiological responses to different environmental stresses in plants. Therefore, metabolomics is an important functional genomics tool that complements genomics and proteomics (Clément et al., 2018;Ghatak et al., 2018). However, metabolomic analysis of sugar beet stress responses has been rare. Using gas chromatography-mass spectrometry (GC-MS), Hossain et al. (2017a) analyzed metabolic changes in the whole leaves and chloroplasts of sugar beet in response to salt stress. Metabolites involved in the Calvin-Benson cycle, glycolysis, and the citric acid cycle exhibited significant decreases in leaves under salt stress. In contrast, the levels of glycolate and serine increased significantly. This result indicates that photorespiratory metabolism is enhanced in the salt-stressed sugar beet. In addition, arabinose, glycolate, inositol, malate, mannitol, and putrescin were found to accumulate in both chloroplasts and extra-chloroplastic space to help maintain the chloroplast biochemical processes through osmotic adjustment. This study also found high levels of the metabolite polyamine putrescine in the chloroplasts, which may play an important role in the acclimation of sugar beet to high salinity stress. The result is consistent with the findings at the transcriptomic level (Ma et al., 2017) and proteomic level (Ji et al., 2019). Since GC-MS profiles primarily central metabolites, liquid chromatography-MS-based untargeted metabolomics approaches (Geng et al., 2016; Geng et al., 2017) may greatly enhance the coverage of the sugar beet metabolome.

## Discussion

**Figure 2:**
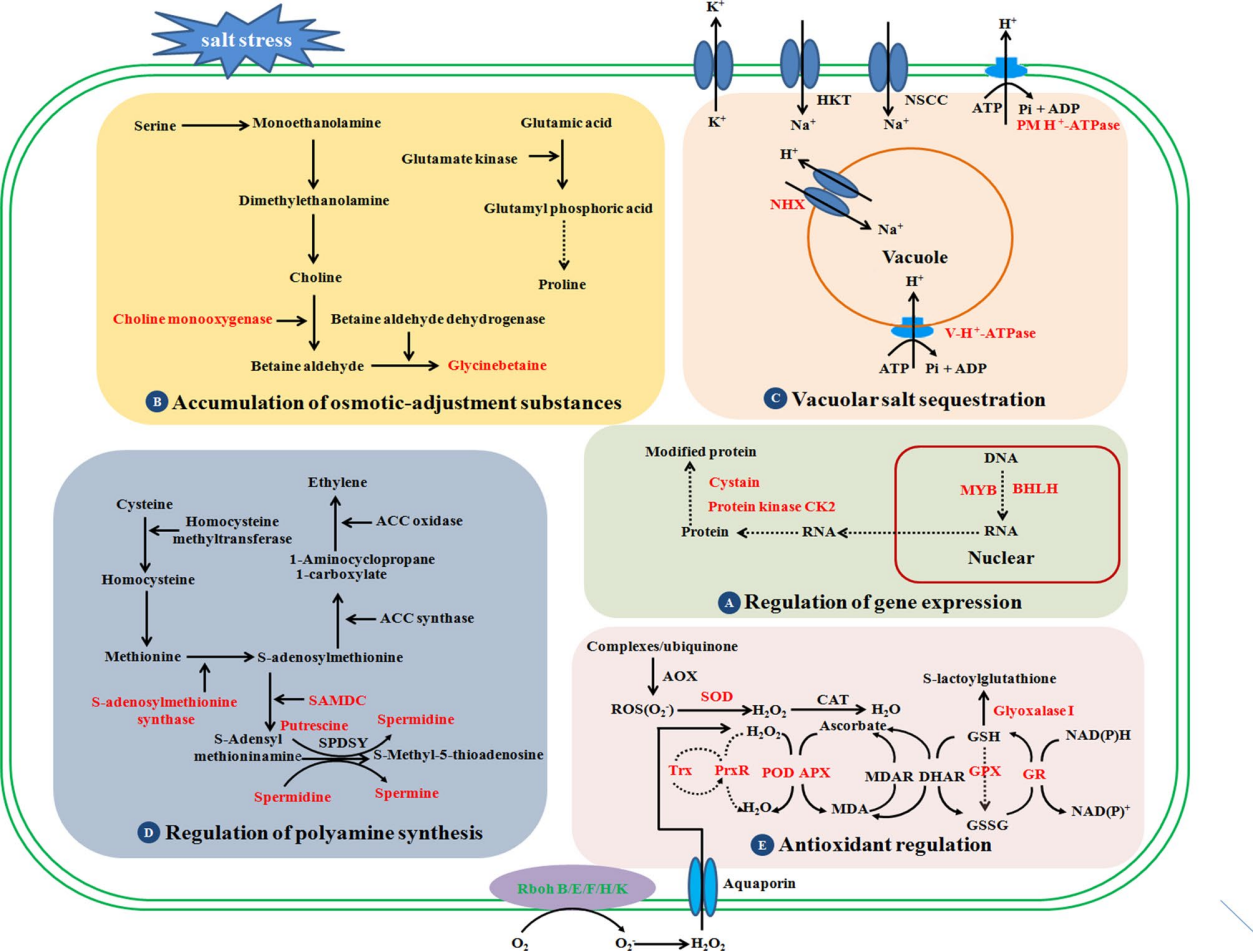
Schematic presentation of the potential mechanisms underlying salt stress tolerance in sugar beet. Red-highlighted genes or proteins play key roles in determining the salt tolerance of sugar beet. Sugar beet can improve its salt tolerance through the following regulation strategies: regulation of gene expression, accumulation of osmotic-adjustment substances, vacuolar salt sequestration, regulation of polyamine synthesis, and antioxidant regulation. NHX, Na^+^/H^+^ antiporters; SOD, superoxide dismutases; Prx, peroxiredoxins; POX, peroxidase; APX, ascorbate peroxidase; CAT, catalase; GR, glutathione reductase; SAMDC, S-adenosylmethionine decarboxylase; RBOH, respiratory burst oxidase homologue; GPX, glutathione peroxidase; CK2, casein kinase; NSCC, non-selective cation channels; HKT, high affinity transporter; GST, glutathione S-transferase, MDAR, monodehydroascorbate reductase; DHAR, dehydroascorbate reductase; MDA, monodehydroascorbate; ROS, reactive oxygen species; AOX, alternative oxidase.

Soil salinization is increasingly problematic and has become a prime concern for global crop production and food security. During evolution, sugar beet has developed various adaptations to combat salt stress, such as osmotic adjustment and osmoregulation, activation of antioxidant defense systems, control of ROS accumulation, and maintenance of ion homeostasis (**Figure 2**). Although sugar beet is more salt-tolerant than other crops, high concentrations of salt significantly affect its yield and quality. There is an immense need to create new sugar beet varieties with stable and high yield in highly saline environments. In the past two decades, several salt tolerance determinants and signaling pathways have been identified in sugar beet, and some of the key candidate genes have been screened for improving sugar beet salt tolerance, such as *CMO*, which is involved in the biosynthesis of GB (**Table 2**, **Figure 2**). Sugar beet salt tolerance is a quantitative trait that is controlled by multiple genes. However, there are few reports on molecular markers and quantitative trait loci (QTLs) associated with salt tolerance. Studies of the salt-overly-sensitive (SOS) pathway and MAPK-related cascades have provided important information about Na^+^ efflux, osmotic, and oxidative stress signaling in the reference plant *Arabidopsis thaliana* (Yang and Guo, 2018a; Yang and Guo, 2018b). Whether these signal transduction pathways play a key role in determining salt tolerance in sugar beet still needs further study. Moreover, many determinants involved in sugar beet tolerance have not been studied in sufficient detail, such as salt-stress signal perception and crosstalk, developmental regulation, and the most important osmotic regulator(s) for salt-tolerance, etc. To date, transcriptomics and proteomics have facilitated our understanding of the nodes and edges in the salt-stress molecular networks, but metabolomics, epigenetics, and multi-omics integrative studies are still lacking. In addition, studies using sugar beet genetic resources (e.g., *Bv*M14) and functional characterization of promising molecular markers for salt-stress tolerance will greatly advance this exciting field of research, which has great potential in agricultural applications.

**Table 2:** Candidate genes for improving salt tolerance in sugar beet.

Candidate genes	Species	Function	Reference
*BvCMO*	*Beta vulgaris*	Synthesis of glycinebetaine	Zhang et al., 2008
*BvSOD*	*B. vulgaris*	Removing accumulation of ROS	Hossain et al., 2017b; Bor et al., 2003
*BvPrx*	*B. vulgaris*	Removing accumulation of ROS	Hossain et al., 2017b; Bor et al., 2003
*BvCAT*	*B. maritime* and *B. vulgaris*	Removing accumulation of ROS	Bor et al., 2003
*BvGR*	*B. maritime* and *B. vulgaris*	Removing accumulation of ROS	Bor et al., 2003
*BvAPX*	*B. maritime* and *B. vulgaris*	Removing accumulation of ROS	Dunajska-Ordak et al., 2014
*BvPOX*	*B. maritime* and *B. vulgaris*	Removing accumulation of ROS	Yolcu et al., 2016
*BvNHX1*	*B. vulgaris*	Vacuolar sequestration of sodium	Adler et al., 2010
*MYB transcription factor*	*B. vulgaris*	Regulating expression of NHX1	Adler et al., 2010
*bHLH transcription factor*	*B. maritime* and *B. vulgaris*	Regulating salt stress response	Skorupa et al., 2019
*PM H* *^+^* *-ATPase*	*B. vulgaris*	Maintaining ion homeostasis	Kirsch et al., 1996; Wakeel et al., 2010
*BvM14-SAMS2*	*Bv*M14	Synthesis of polyamines	Ma et al., 2017
*BvM14-SAMDC*	*Bv*M14	Synthesis of polyamines	Ji et al., 2019
*BvM14-glyoxalase I*	*Bv*M14	Detoxifying methylglyoxal	Wu et al., 2013b
*BvM14-cystatin*	*Bv*M14	Increasing salt tolerance	Wang et al., 2012
*BvCK2*	*B. vulgaris*	Signal transduction and stress response	Kanhonou et al., 2001

NHX, Na^+^/H^+^ antiporters; SOD, superoxide dismutases; Prx, peroxiredoxins; POX, peroxidase; APX, ascorbate peroxidase; CAT, catalase; GR, glutathione reductase; SAMDC, S-adenosylmethionine decarboxylase; SAMS, S-adenosylmethionine synthetase; GPX, glutathione peroxidase; CK2, casein kinase; ROS, reactive oxygen species; AOX, alternative oxidase; CMO, choline monooxygenase.

## Author Contributions

YW, XL, and SC wrote and revised the manuscript. All authors read and approved the final manuscript.

## Funding

This research was support by the National Natural Science Foundation of China Project (31701487), Basic Research Work Program of Heilongjiang Provincial Higher Education Institutions (KJCXYB201706), and Youth Innovative Talents Training Program of Heilongjiang Regular Universities.

## Conflict of Interest

The authors declare that the research was conducted in the absence of any commercial or financial relationships that could be construed as a potential conflict of interest.

## References

[B1] AboKassem E. E. M. (2007). Effects of salinity: calcium interaction on growth and nucleic acid metabolism in five spices of *Chenopodiaceae*. Turk. J. Bot. 31, 125–134.

[B2] Adler G. Blumwald E. Bar-Zvi D. (2010). The sugar beet gene encoding the sodium/proton exchanger 1 (*BvNHX1*) is regulated by a MYB transcription factor. Planta. 232, 187–195. 10.1007/s00425-010-1160-7 20390294 PMC2872020

[B3] Apel K. Hirt H. (2004). Reactive oxygen species: metabolism, oxidative stress, and signal transduction. Annu. Rev. Plant Biol. 55, 373–399. 10.1016/j.jplph.2014.08.022 15377225

[B4] Baldwin J. C. Dombrowski J. E. (2006). Evaluation of *Lolium temulentum* as a model grass species for the study of salinity stress by PCR-based subtractive suppression hybridization analysis. Plant Sci. 171, 459–469. 10.1016/j.plantsci.2006.05.003 25193643

[B5] Baxter A. Mittler R. Suzuki N. (2014). ROS as key players in plant stress signaling. J. Exp. Bot. 65, 1229–1240. 10.1093/jxb/ert375 24253197

[B6] Ben Rejeb K. Benzarti M. Debez A. Bailly C. Savouré A. Abdelly C. (2015). NADPH oxidase-dependent H_2_O_2_ production is required for salt-induced antioxidant defense in *Arabidopsis thaliana*. J. Plant Physiol. 174, 5–15. 10.1016/j.jplph.2014.08.022 25462961

[B7] Blumwald E. (2000). Sodium transport and salt tolerance in plants. Curr. Opin. Cell Biol. 12, 431–434. 10.1016/S0955-0674(00)00112-5 10873827

[B8] Blumwald E. Poole R. J. (1987). Salt tolerance in suspension cultures of sugar beet: induction of Na^+^/H^+^ antiport activity at the tonoplast by growth in salt. Plant Physiol. 83, 884–887. 10.1104/pp.83.4.884 16665356 PMC1056467

[B9] Bor M. Özdemir F. Türkan I. (2003). The effect of salt stress on lipid peroxidation and antioxidants in leaves of sugar beet *Beta vulgaris* L. and wild beet *Beta maritime* L. Plant Sci. 164, 77–84. 10.1016/S0168-9452(02)00338-2

[B10] Bushman B. S. Amundsen K. L. Warnke S. E. Robins J. G. Johnson P. G. (2016). Transcriptome profiling of Kentucky bluegrass (*Poa pratensis* L.) accessions in response to salt stress. BMC Genomics 17, 48. 10.1186/s12864-016-2379-x 26758626 PMC4711080

[B11] Chamizo-Ampudia A. Sanz-Luque E. Llamas A. Galvan A. Fernandez E. (2017). Nitrate reductase regulates plant nitric oxide homeostasis. Trends Plant Sci. 22, 163–174. 10.1016/j.tplants.2016.12.001 28065651

[B12] Chen S. Harmon A. C. (2006). Advances in plant proteomics. Proteomics. 6, 5504–5516. 10.1002/pmic.200600143 16972296

[B13] Chen T. H. Murata N. (2002). Enhancement of tolerance of abiotic stress by metabolic engineering of betaines and other compatible solutes. Curr. Opin. Plant Biol. 5, 250–257. 10.1016/S1369-5266(02)00255-8 11960744

[B14] Clément G. Moison M. Soulay F. Reisdorf-Cren M. Masclaux-Daubresse C. (2018). Metabolomics of laminae and midvein during leaf senescence and source-sink metabolite management in *Brassica napus* L. leaves. J. Exp. Bot. 69, 891–903. 10.1093/jxb/erx253 28992054 PMC5853214

[B15] Daoud S. Koyro H. W. Harrouni M. C. Schmidt A. Papenbrock J. (2003). “Salinity tolerance of *Beta vulgaris* ssp.*maritima.* Part II. Physiological and biochemical regulation,” in Cash crop halophytes: recent studies. Tasks for Vegetation Science. Eds. Lieth H. Mochtchenko M. (Dordrecht: Springer press).

[B16] De la Torre-González A. Montesinos-Pereira D. Blasco B. Ruiz J. M. (2018). Influence of the proline metabolism and glycine betaine on tolerance to salt stress in tomato (*Solanum lycopersicum* L.) commercial genotypes. J. Plant Physiol. 231, 329–336. 10.1016/j.jplph.2018.10.013 30388672

[B17] Deinlein U. Stephan A. B. Horie T. Luo W. Xu G. Schroeder J. I. (2014). Plant salt-tolerance mechanisms. Trends Plant Sci. 19, 371–379. 10.1016/j.tplants.2014.02.001 24630845 PMC4041829

[B18] Dohm J. C. Minoche A. E. Holtgräwe D. Capella-Gutiérrez S. Zakrzewski F. Tafer H. (2013). The genome of the recently domesticated crop plant sugar beet (*Beta vulgaris*). Nature. 23, 546–5499. 10.1038/nature12817 24352233

[B19] Doney D. L. Whitney E. D. Terry J. Frese L. Fitzgerald P. (1999). The distribution and dispersal of *Beta vulgaris* L. ssp. *maritima* germplasm in England, Wales, and Ireland. J. Sugar Beet Res. 27, 29–37. 10.5274/jsbr.27.1.29

[B20] Dunajska-Ordak K. Skorupa-Kłaput M. Kurnik K. Tretyn A. Tyburski J. (2014). Cloning and expression analysis of a gene encoding for ascorbate peroxidase and responsive to salt stress in beet (*Beta vulgaris*). Plant Mol. Biol. Rep. 32, 162–175. 10.1007/s11105-013-0636-6 24465083 PMC3893476

[B21] Geng S. Misra B. B. de Armas E. Huhman D. V. Alborn H. T. Sumner L. W. (2016). Jasmonate-mediated stomatal closure under elevated CO_2_ revealed by time-resolved metabolomics. Plant J. 88, 947–962. 10.1111/tpj.13296 27500669

[B22] Geng S. Yu B. Zhu N. Dufresne C. Chen S. (2017). Metabolomics and proteomics of *Brassica napus* guard cells in response to low CO_2_. Front. Mol. Biosci. 4, 51. 10.3389/fmolb.2017.00051 28791296 PMC5525006

[B23] Ghatak A. Chaturvedi P. Weckwerth W. (2018). Metabolomics in plant stress physiology. Adv. Biochem. Eng. Biotechnol. 164, 187–236. 10.1007/10-2017-55 29470599

[B24] Ghoulam C. Foursy A. Fares K. (2002). Effects of salt stress on growth, inorganic ions and proline accumulation in relation to osmotic adjustment in five sugar beet cultivars. Environ. Exp. Bot. 47, 39–50. 10.1016/s0098-8472(01)00109-5

[B25] Gong Q. Li P. Ma S. Indu Rupassara S. Bohnert H. J. (2010). Salinity stress adaptation competence inthe extremophile *Thellungiella halophila* in comparison with its relative *Arabidopsis thaliana* . Plant J. 44, 826–839. 10.1111/j.1365-313X.2005.02587.x 16297073

[B26] Guan Q. Wang Z. Wang X. Takano T. Liu S. (2015). A peroxisomal APX from *Puccinellia tenuiflora* improves the abiotic stress tolerance of transgenic *Arabidopsis thaliana* through decreasing of H_2_O_2_ accumulation. J. Plant Physiol. 175, 183–191. 10.1016/j.jplph.2014.10.020 25644292

[B27] Hajiboland R. Joudmand A. Fotouhi K. (2009). Mild salinity improves sugar beet (*Beta vulgaris* L.) quality. Acta Agric. Scand. Sect. B-Soil Plant Sci. 59, 295–305. 10.1080/09064710802154714

[B28] Hibino T. Waditee R. Araki E. Ishikawa H. Aoki K. Tanaka Y. (2002). Functional characterization of choline monooxygenase, an enzyme for betaine synthesis in plants. J. Biol. Chem. 277, 41352–41360. 10.1074/jbc.M205965200 12192001

[B29] Hossain M. S. Persicke M. Elsayed A. I. Kalinowski J. Dietz K. J. (2017a). Metabolite profiling at the cellular and subcellular level reveals metabolites associated with salinity tolerance in sugar beet. J. Exp. Bot. 68, 5961–5976. 10.1093/jxb/erx388 29140437 PMC5854137

[B30] Hossain M. S. Elsayed A. I. Moore M. Dietz K. J. (2017b). Redox and reactive oxygen species network in acclimation for salinity tolerance in sugar beet. J. Exp. Bot. 68, 1283–1298. 10.1093/jxb/erx019 28338762 PMC5441856

[B31] Hubbard M. J. Cohen P. (1993). On target with a new mechanism for the regulation of protein phosphorylation. Trends Biochem. Sci. 18, 172–177. 10.1016/0968-0004(93)90109-Z 8392229

[B32] Jafarzadeh A. A. Aliasgharzad N. (2007). Salinity and salt composition effects on seed germination and root length of four sugar beet cultivars. Biologia. 62, 562–564. 10.2478/s11756-007-0111-7

[B33] Jha U. C. Bohra A. Jha R. Parida S. K. (2019). Salinity stress response and ‘omics’ approaches for improving salinity stress tolerance in major grain legumes. Plant Cell Rep. 38, 255–277. 10.1007/s00299-019-02374-5 30637478

[B34] Ji M. Wang K. Wang L. Chen S. Li H. Ma C. (2019). Overexpression of an *S*-adenosylmethionine decarboxylase from sugar beet m14 increased *Arabidopsis* salt tolerance. Int. J. Mol. Sci. 23, 20. 10.3390/ijms20081990E1990. PMC6515516 31018555

[B35] Kaffka S. Hembree K. (2004). The effects of saline soil, irrigation, and seed treatment on sugar beet stand establishment. J. Sugar Beet Res. 41, 61–72. 10.5274/jsbr.41.3.61

[B36] Kanhonou R. Serrano R. Palau R. R. (2001). A catalytic subunit of the sugar beet protein kinase CK2 is induced by salt stress and increases NaCl tolerance in *Saccharomyces cerevisiae* . Plant Mol. Biol. 47, 571–579. 10.1023/a:1012227913356 11725943

[B37] Katerji N. Van Hoorn J. W. Hamdy A. Mastrorilli M. Karzel E. M. (1997). Osmotic adjustment of sugar beets in response to soil salinity and its influence on stomatal conductance, growth and yield. Agric. Water Manage. 34, 57–69. 10.1016/S0378-3774(96)01294-2

[B38] Khayamim S. Afshari R. T. Sadeghian S. Y. Poustini K. Rouzbeh F. Abbasi Z. (2014). Seed germination, plant establishment, and yield of sugar beet genotypes under salinity stress. J. Agric. Sci. Technol. 16, 779–790. 10.1016/j.biosystemseng.2014.05.006

[B39] Kirsch M. An Z. Viereck R. Löw R. Rausch T. (1996). Salt stress induces an increased expression of V-type H^+^-ATPase in mature sugar beet leaves. Plant Mol. Biol. 32, 543–547. 10.1007/BF00019107 8980504

[B40] Lei Y. Xu Y. Hettenhausen C. Lu C. Shen G. Zhang C. (2018). Comparative analysis of alfalfa (*Medicago sativa* L.) leaf transcriptomes reveals genotype-specific salt tolerance mechanisms. BMC Plant Biol. 18, 35. 10.1186/s12870-018-1250-4 29448940 PMC5815232

[B41] Li H. Cao H. Wang Y. Pang Q. Ma C. Chen S. (2010). Proteomic analysis of sugar beet apomictic monosomic addition line M14. J. Proteomics 73, 297–308. 10.1016/j.jprot.2009.09.012 19782777

[B42] Li H. Pan Y. Zhang Y. Wu C. Ma C. Yu B. (2015). Salt stress response of membrane proteome of sugar beet monosomic addition line M14 . J. Proteomics 127, 18–33. 10.1016/j.jprot.2015.03.025 25845583

[B43] Liang W. Ma X. Wan P. Liu L. (2018). Plant salt-tolerance mechanism: a review. Biochem. Biophys. Res. Commun. 495, 286–291. 10.1016/j.bbrc.2017.11.043 29128358

[B44] Liu H. Wang Q. Yu M. Zhang Y. Wu Y. Zhang H. (2010). Transgenic salt-tolerant sugar beet (*Beta vulgaris* L.) constitutively expressing an *Arabidopsis thaliana* vacuolar Na^+^/H^+^ antiporter gene, *AtNHX3*, accumulates more soluble sugar but less salt in storage roots. Plant Cell Environ. 31, 1325–1334. 10.1111/j.1365-3040.2008.01838.x 18518917

[B45] Lv X. Jin Y. Wang Y. (2018). De novo, transcriptome assembly and identification of salt-responsive genes in sugar beet M14. Comput. Biol. Chem. 75, 1–10. 10.1016/j.compbiolchem.2018.04.014 29705503

[B46] Ma C. Wang Y. Gu D. Nan J. Chen S. Li H. (2017). Overexpression of *S*-adenosyl-l-methionine synthetase 2 from sugar beet M14 increased *Arabidopsis* tolerance to salt and oxidative stress. Int. J. Mol. Sci. 18, E847. 10.3390/ijms18040847 PMC5412431 28420190

[B47] Magaña C. Núñez-Sánchez N. Fernández-Cabanás V. M. García P. Serrano A. Pérez-Marín D. (2011). Direct prediction of bioethanol yield in sugar beet pulp using near infrared spectroscopy. Bioresour. Technol. 102, 9542–9549. 10.1016/j.biortech.2011.07.045 21872469

[B48] Marschner H. (1995). Mineral nutrition of higher plants. London, UK: Academic Press.

[B49] Mittler R. Vanderauwera S. Gollery M. Van Breusegem F. (2004). Reactive oxygen gene network of plants. Trends Plant Sci. 9, 490–498. 10.1016/j.tplants.2004.08.009 15465684

[B50] Munns R. Tester M. (2008). Mechanisms of salinity tolerance. Annu. Rev. Plant Biol. 59, 651–681. 10.1146/annurev.arplant.59.032607.092911 18444910

[B51] Nadeem M. Li J. Yahya M. Wang M. Ali A. Cheng A. (2019). Grain legumes and fear of salt stress: focus on mechanisms and management strategies. Int. J. Mol. Sci. 20, E799. 10.3390/ijms20040799 PMC6412900 30781763

[B52] Olías R. Eljakaoui Z. Li J. De Morales P. A. Marín-Manzano M. C. Pardo J. M. (2009). The plasma membrane Na^+^/H^+^ antiporter SOS1 is essential for salt tolerance in tomato and affects the partitioning of Na^+^ between plant organs. Plant Cell Environ. 32, 904–916. 10.1111/j.1365-3040.2009.01971.x 19302170

[B53] Ouyang B. Yang T. Li H. Zhang L. Zhang Y. Zhang J. (2006). Identification of early salt stress response genes in tomato root by suppression subtractive hybridization and microarray analysis. J. Exp. Bot. 58, 507–520. 10.1093/jxb/erl258 17210988

[B54] Per T. S. Khan N. A. Reddy P. S. Masood A. Hasanuzzaman M. Khan M. I. R. (2017). Approaches in modulating proline metabolism in plants for salt and drought stress tolerance: phytohormones, mineral nutrients and transgenics. Plant Physiol. Biochem. 115, 126–140. 10.1016/j.plaphy.2017.03.018 28364709

[B55] Pradedova E. V. Nimaeva O. D. Saliaev R. K. (2014). Effect of stress conditions on the activity and isozyme composition of peroxidase of vacuoles and tissue extract of red beet roots. Izv. Akad. Nauk. Ser. Biol. 41, 254–263. 10.1134/S106235901403008X 25731036

[B56] Russell B. L. Rathinasabapathi B. Hanson A. D. (1998). Osmotic stress induces expression of choline monooxygenase in sugar beet and amaranth. Plant Physiol. 116, 859–865. 10.2307/4278159 9489025 PMC35146

[B57] Sadeghian S. Y. Yavari N. (2010). Effect of water-deficit stress on germination and early seedling growth in sugar beet. J. Agron. Crop Sci. 190, 138–144. 10.1111/j.1439-037X.2004.00087.x

[B58] Sadughi M. Sharifan H. Pessarakli M. (2015). Effects of caspian sea water on sugar beet seed germination. J. Plant Nutr. 38, 1685–1693. 10.1080/01904167.2015.1042164

[B59] Shabala L. Zhang J. Pottosin I. Bose J. Zhu M. Fuglsang A. T. (2016). Cell-type-specific H^+^-ATPase activity in root tissues enables K^+^ retention and mediates acclimation of barley (*Hordeum vulgare*) to salinity stress. Plant Physiol. 172, 2445–2458. 10.1104/pp.16.01347 27770060 PMC5129721

[B60] Shabala S. Wu H. Bose J. (2015). Salt stress sensing and early signaling events in plant roots: current knowledge and hypothesis. Plant Sci. 241, 109–119. 10.1016/j.plantsci.2015.10.003 26706063

[B61] Skorupa M. Gołębiewski M. Domagalski K. Kurnik K. Abu Nahia K. Złoch M. (2016). Transcriptomic profiling of the salt stress response in excised leaves of the halophyte *Beta vulgaris* ssp. Marit. Plant Sci. 243, 56–70. 10.1016/j.plantsci.2015.11.007 26795151

[B62] Skorupa M. Gołębiewski M. Kurnik K. Niedojadło J. Kęsy J. Klamkowski K. (2019). Salt stress vs. salt shock-the case of sugar beet and its halophytic ancestor. BMC Plant Biol. 19, 57. 10.1186/s12870-019-1661-x 30727960 PMC6364445

[B63] Sun J. Wang M. J. Ding M. Q. Deng S. R. Liu M. Q. Lu C. F. (2010). H_2_O_2_ and cytosolic Ca^2+^ signals triggered by the PM H^+^-coupled transport system mediate K^+^/Na^+^ homeostasis in NaCl-stressed *Populus euphratica* cells. Plant Cell Environ. 33, 943–958. 10.1111/j.1365-3040.2010.02118.x 20082667

[B64] Sun X. Wang Y. Xu L. Li C. Zhang W. Luo X. (2017). Unraveling the root proteome changes and its relationship to molecular mechanism underlying salt stress response in Radish (*Raphanus sativus* L.). Front. Plant Sci. 8, 1192. 10.3389/fpls.2017.01192 28769938 PMC5509946

[B65] Sun Y. Wang F. Wang N. Dong Y. Liu Q. Zhao L. (2013). Transcriptome exploration in *Leymus chinensis* under saline-alkaline treatment using 454 pyrosequencing. PloS One 8, e53632. 10.1371/journal.pone.0053632 PMC3554714 23365637

[B66] Tanou G. Filippou P. Belghazi M. Job D. Diamantidis G. Fotopoulos V. (2012). Oxidative and nitrosative-based signaling and associated post-translational modifications orchestrate the acclimation of citrus plants to salinity stress. Plant J. 72, 585–599. 10.1111/j.1365-313X.2012.05100.x 22780834

[B67] Waditee R. Bhuiyan N. H. Hirata E. Hibino T. Tanaka Y. Shikata M. (2007). Metabolic engineering for betaine accumulation in microbes and plants. J. Biol. Chem. 282, 34185–34193. 10.1074/jbc.M704939200 17884813

[B68] Wakeel A. Hanstein S. Pitann B. Schubert S. (2010). Hydrolytic and pumping activity of H^+^-ATPase from leaves of sugar beet (*Beta vulgaris* L.) as affected by salt stress. J. Plant Physiol. 167, 725–731. 10.1016/j.jplph.2009.12.018 20189265

[B69] Wakeel A. Asif A. R. Pitann B. Schubert S. (2011a). Proteome analysis of sugar beet (*Beta vulgaris* L.) elucidates constitutive adaptation during the first phase of salt stress. J. Plant Physiol. 168, 519–526. 10.1016/j.jplph.2010.08.016 20980072

[B70] Wakeel A. Sümer A. Hanstein S. Yan F. Schubert S. (2011b). In vitro effect of different Na^+^/K^+^ ratios on plasma membrane H^+^-ATPase activity in maize and sugar beet shoot. Plant Physiol. Biochem. 49, 341–345. 10.1016/j.plaphy.2011.01.006 21282062

[B71] Walley J. W. Sartor R. C. Shen Z. Schmitz R. J. Wu K. J. Urich M. A. (2016). Integration of omic networks in a developmental atlas of maize. Science. 353, 814–818. 10.1126/science.aag1125 27540173 PMC5808982

[B72] Wang X. L. He R. F. He G. C. (2005). Construction of suppression subtractive hybridization libraries and identification of brown plant hopper-induced genes. J. Plant Physiol. 162, 1254–1262. 10.1016/j.jplph.2005.01.005 16323277

[B73] Wang Y. Stevanato P. Yu L. Zhao H. Sun X. Sun F. (2017). The physiological and metabolic changes in sugar beet seedlings under different levels of salt stress. J. Plant Res. 130, 1079–1093. 10.1007/s10265-017-0964-y 28711996

[B74] Wang Y. Stevanato P. Lv C. Li R. Geng G. (2019). Comparative physiological and proteomic analysis of two sugar beet genotypes with contrasting salt tolerance. J. Agric. Food Chem. 67, 6056–6073. 10.1021/acs.jafc.9b00244 31070911

[B75] Wang Y. Zhan Y. Wu C. Gong S. Zhu N. Chen S. (2012). Cloning of a cystatin gene from sugar beet M14 that can enhance plant salt tolerance. Plant Sci. 191-192, 93–99. 10.1016/j.plantsci.2012.05.001 22682568

[B76] Ward J. M. Hirschi K. D. Sze H. (2003). Plants pass the salt. Trends Plant Sci. 8, 200–201. 10.1016/s1360-1385(03)00059-1 12758034

[B77] Wu G. Liang N. Feng R. Zhang J. (2013a). Evaluation of salinity tolerance in seedlings of sugar beet (*Beta vulgaris* L.) cultivars using proline, soluble sugars and cation accumulation criteria. Acta Physiol. Plant 35, 2665–2674. 10.1007/s11738-013-1298-6

[B78] Wu C. Ma C. Pan Y. Gong S. Zhao C. Chen S. (2013b). Sugar beet M14 glyoxalase I gene can enhance plant tolerance to abiotic stresses. J. Plant Res. 126, 415–425. 10.1007/s10265-012-0532-4 23203352

[B79] Wu G. Feng R. Liang N. Yuan H. Sun W. (2015a). Sodium chloride stimulates growth and alleviates sorbitol-induced osmotic stress in sugar beet seedlings. Plant Growth Regul. 75, 307–316. 10.1007/s10725-014-9954-4

[B80] Wu G. Shui Q. Wang C. Zhang J. Yuan H. Li S. (2015b). Characteristics of Na^+^ uptake in sugar beet (*Beta vulgaris* L.) seedlings under mild salt conditions. Acta Physiol. Plant 37, 70. 10.1007/s11738-015-1816-9

[B81] Wu G. Wang J. Li S. (2019). Genome-wide identification of Na^+^/H^+^ antiporter (NHX) genes in sugar beet (*Beta vulgaris* L.) and their regulated expression under salt stress. Genes. 10, E401. 10.3390/genes10050401 PMC6562666 31137880

[B82] Xia T. Apse M. P. Aharon G. S. Blumwald E. (2002). Identification and characterization ofa NaCl-inducible vacuolar Na^+^/H^+^ antiporter in *Beta vulgaris* . Physiol. Plant 116, 201–212. 10.1034/j.1399-3054.2002.1160210.x 12354197

[B83] Yamada N. Promden W. Yamane K. Tamagake H. Hibino T. Tanaka Y. (2009). Preferential accumulation of betaine uncoupled to choline monooxygenase in young leaves of sugar beet-importance of long-distance translocation of betaine under normal and salt-stressed conditions. J. Plant Physiol. 166, 2058–2070. 10.1016/j.jplph.2009.06.016 19647889

[B84] Yamada N. Sakakibara S. Tsutsumi K. Waditee R. Tanaka Y. Takabe T. (2011). Expression and substrate specificity of betaine/proline transporters suggest a novel choline transport mechanism in sugar beet. J. Plant Physiol. 168, 1609–1616. 10.1016/j.jplph.2011.03.007 21511362

[B85] Yamada N. Takahashi H. Kitou K. Sahashi K. Tamagake H. Tanaka Y. (2015). Suppressed expression of choline monooxygenase in sugar beet on the accumulation of glycinebetaine. Plant Physiol. Biochem. 96, 217–221. 10.1016/j.plaphy.2015.06.014 26302482

[B86] Yang A. Duan X. Gu X. Gao F. Zhang J. (2005). Efficient transformation of beet (*Beta vulgaris*) and production of plants with improved salt-tolerance. Plant Cell Tissue Organ Cult. 83, 259–270. 10.1007/s11240-005-6670-9

[B87] Yang L. Ma C. Wang L. Chen S. Li H. (2012). Salt stress induced proteome and transcriptome changes in sugar beet monosomic addition line M14. J. Plant Physiol. 169, 839–850. 10.1016/j.jplph.2012.01.023 22498239

[B88] Yang L. Zhang Y. Zhu N. Koh J. Ma C. Pan Y. (2013). Proteomic analysis of salt tolerance in sugar beet monosomic addition line m14. J. Proteome Res. 12, 4931–4950. 10.1021/pr400177m 23799291

[B89] Yang Y. Guo Y. (2018a). Unraveling salt stress signaling in plants. J. Integr. Plant Biol. 60, 796–804. 10.1111/jipb.12689 29905393

[B90] Yang Y. Guo Y. (2018b). Elucidating the molecular mechanisms mediating plant salt-stress responses. New Phytol. 217, 523–539. 10.1111/nph.14920 29205383

[B91] Yokoi S. Quintero F. J. Cubero B. Ruiz M. T. Bressan R. A. Hasegawa P. M. (2002). Differential expression and function of *Arabidopsis thaliana* NHX Na^+^/H^+^ antiporters in the salt stress response. Plant J. 30, 529–539. 10.1046/j.1365-313X.2002.01309.x 12047628

[B92] Yolcu S. Ozdemir F. Güler A. Bor M. (2016). Histone acetylation influences the transcriptional activation of Pox in *Beta vulgaris* L. and *Beta maritima* L. under salt stress. Plant Physiol. Biochem. 100, 37–46. 10.1016/j.plaphy.2015.12.019 26773543

[B93] Yu B. Li J. Koh J. Dufresne C. Yang N. Qi S. (2016). Quantitative proteomics and phosphoproteomics of sugar beet monosomic addition line m14 in response to salt stress. J. Proteomics. 143, 286–297. 10.1016/j.jprot.2016.04.011 27233743

[B94] Zhang J. L. Shi H. (2013). Physiological and molecular mechanisms of plant salt tolerance. Photosyn. Res. 115, 1–22. 10.1007/s11120-013-9813-6 23539361

[B95] Zhang J. Tan W. Yang X. H. Zhang H. X. (2008). Plastid-expressed choline monooxygenase gene improves salt and drought tolerance through accumulation of glycine betaine in tobacco. Plant Cell Rep. 27, 1113–1124. 10.1007/s00299-008-0549-2 18437388

[B96] Zhou J. Jiao F. Wu Z. Li Y. Wang X. He X. (2008). OsPHR2 is involved in phosphate-starvation signaling and excessive phosphate accumulation in shoots of plants. Plant Physiol. 146, 1673–1686. 10.1104/pp.107.111443 18263782 PMC2287342

[B97] Zhu J. K. (2016). Abiotic stress signaling and responses in plants. Cell. 167, 313–324. 10.1016/j.cell.2016.08.029 27716505 PMC5104190

[B98] Zhu J. K. (2003). Regulation of ion homeostasis under salt stress. Curr. Opin. Plant Biol. 6, 441–445. 10.1016/S1369-5266(03)00085-2 12972044

